# Supplementary material for: CircDCLRE1C Regulated Lipopolysaccharide-Induced Inflammatory Response and Apoptosis by Regulating miR-214b-3p/STAT3 Pathway in Macrophages
Source: Int J Mol Sci. 2022 Jun 19;23(12):6822. doi: 10.3390/ijms23126822 (PMC9224735; doi:10.3390/ijms23126822)
Supplement: Supplementary file 1 [file ijms-23-06822-s001.zip › ijms-1710003-supplementary.pdf]

Supplementary material

1. Supplementary Figures

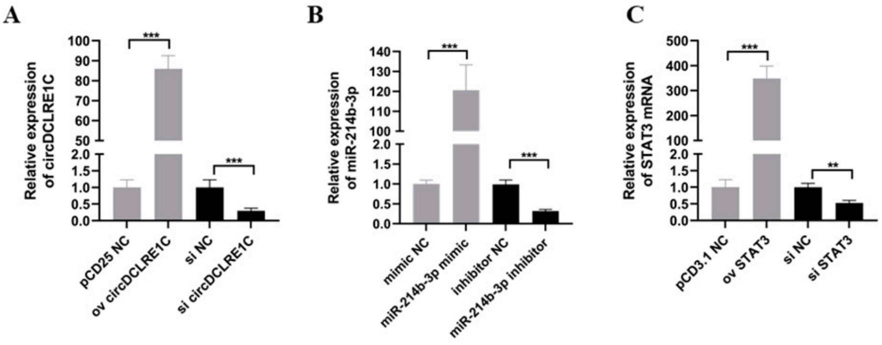

Supplementary Figure S1. Transfection efficiency detection. (A, B, C) The efficiency of overexpression and knockdown of circDCLRE1C, miR-214b-3p and STAT3 in HD11

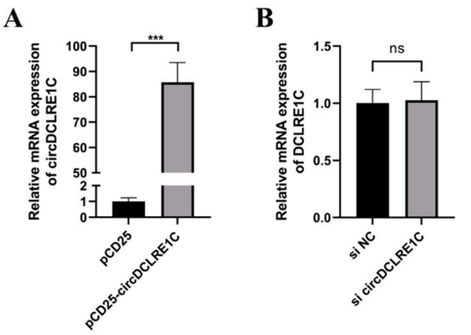

Supplementary Figure S2. The reliable detection of circDCLRE1C overexpression and knockdown. (A) qRT-PCR analysis for the detection of circDCLRE1C expression in HD11 cells transfected with pCD25-circDCLRE1C plasmid and treated with RNase R. (B) The expression of DCLRE1C in HD11 cells transfected with si-circDCLRE1C was detected by qRT-PCR.

2. Supplementary Tables

Supplementary Table S1. The sequence of circDCLRE1C

circRNA name	The sequence information
circDCLRE1C	GTGCATGTGAATAAGCTTGATATGTTCAAAAATATGCCAGAAATCCT<br>GTACCATATAACTACAGACCGATACACTCAGATTCATGCCTGTCGAC<br>ATCCCAAGGACGATGACTACGTTCGAGGGAACAGACTGCCCTGTG<br>GAATCACTTGCCAAAATGGAACTCCCTTGCACGTAATTAGCATCAA<br>ACCTTCCACTATGTGGTTTGGAGAAAGGATCAAGAAAACCAATGTA<br>ATAGTGAG

Supplementary Table S2. The primers used in this study

Primer name	Fragment sequence (5'-3')	Application
DCLRE1C-F	GCTACAGAGCAAACACAGCTT	Q-PCR
DCLRE1C-R	GACTCCTGCTGGTTGCTAGG	Q-PCR
q-circDCLRE1C-F	AATCACTTGCCAAAATGGAAC	Q-PCR
q-circDCLRE1C-R	GTTCCATTTTGGCAAGTGATT	Q-PCR
STAT3-F	GGCAGCAGATCAAAGCCCTG	Q-PCR
STAT3-R	GCAGCGTATGCCCAGTCC	Q-PCR
IL-6-F	CGATCCGGCAGATGGTGATA	Q-PCR
IL-6-R	CAGAGGATTGTGCCCGAACT	Q-PCR
TNF-α-F	CGCTCAGAACGACGTCAA	Q-PCR
TNF-α-R	GTCGTCCACACCAACGAG	Q-PCR
IFN-γ-F	CTGACAAGTCAAAGCCGCAC	Q-PCR
IFN-γ-R	TCAAGTCGTTCATCGGGAGC	Q-PCR
Actin-F	TTGTTGACAATGGCTCCGGT	Q-PCR
Actin-R	AACCATCACACCCTGATGTCT	Q-PCR

Supplementary Table S3. RNA oligonucleotides sequence information

Sequence name	Fragment sequence (5'-3')	Application
si-circDCLRE1C	GTGAGGTGCATGTGAATAA	circDCLRE1C knockdown
siSTAT3	GGACATCAGTGGAAAGACT	STAT3 knockdown
miR-214b-3p inhibitor	CACAGCAAGUGUAGACAGGCA	miR-214b-3p knockdown
